# Arthroscopic resection of a lateral bipartite talus with anterior talofibular ligament capsuloligamentous repair: a rare case report

**DOI:** 10.1097/RC9.0000000000000940

**Published:** 2026-09-14

**Authors:** Andi Praja Wira Yudha Luthfi, Filbert Nathaniel Winoto, Karina Sylvana Gani, Mitchel Mitchel, Erica Kholinne

**Affiliations:** a Department of Orthopedic and Traumatology, Bhayangkara Tk.I Pusdokkes Polri Hospital, Jakarta, Indonesia; b Department of Orthopedic and Traumatology, Gatam Institute, Eka Hospital, Tangerang, Indonesia; c Faculty of Medicine and Health Sciences, Atma Jaya Catholic University of Indonesia, Jakarta, Indonesia; d Department of Surgery, Faculty of Medicine, Universitas Trisakti, Jakarta, Indonesia

**Keywords:** anterior talofibular ligament, arthroscopy, bipartite talus, Broström repair, talus bipartitus

## Abstract

**Background:**

Bipartite talus (talus bipartitus) is a rare anatomical variant characterized by separation of the talus into two fragments. Most reported cases involve the posterior talar process, whereas a lateral bipartite talus is exceptionally uncommon.

**Case presentation:**

A 42-year-old man presented with chronic lateral ankle pain and a palpable bony prominence. Radiographs and computed tomography demonstrated a well-corticated fragment separated from the talar body, consistent with a bipartite talus. Arthroscopic excision of the fragment was performed. Intraoperatively, the anterior talofibular ligament (ATFL) was found to be attached to the fragment; therefore, arthroscopic Broström capsuloligamentous repair using an all-suture anchor was subsequently performed to restore lateral ankle stability. At the 18-month follow-up, the patient demonstrated marked symptomatic improvement with restoration of daily activities, improved functional scores, and no evidence of residual fragment or postoperative instability.

**Discussion:**

A bipartite talus may mimic a fracture nonunion or an accessory ossicle, making advanced imaging important for diagnosis. In this case, the fragment was located laterally, with involvement of the ATFL footprint. Arthroscopy enabled minimally invasive fragment excision and simultaneous ligament repair.

**Conclusion:**

Lateral bipartite talus is an exceptionally rare condition that may mimic other fracture-related conditions and compromise lateral ankle stability because of the ATFL attachment. Arthroscopic fragment excision combined with ATFL capsuloligamentous repair was a feasible, minimally invasive treatment with favorable clinical outcomes.

## Introduction

Bipartition is a condition in which a single bone is divided into two distinct fragments, usually connected by fibrous, cartilaginous, or partially ossified tissue. A bipartite talus is an exceptionally rare anatomical variant, most commonly involving the posterior talar process and frequently presenting with posterior ankle or subtalar symptoms. Although many anatomical variations of the ankle and foot are discovered incidentally on imaging, a bipartite talus may become symptomatic and lead to pain, restricted joint motion, or degenerative changes involving the ankle or subtalar joint^[^1–3^]^.

HIGHLIGHTS

- A bipartite talus is a rare congenital anomaly that may mimic fracture nonunion or accessory ossicles.
- A lateral bipartite talus with attachment of the anterior talofibular ligament (ATFL) is exceptionally uncommon.
- Computed tomography was useful in identifying the well-corticated fragment and differentiating it from an acute fracture.
- Arthroscopic excision enabled minimally invasive removal of the symptomatic fragment with minimal soft-tissue disruption.
- Arthroscopic Broström capsuloligamentous repair successfully restored lateral ankle stability with favorable 18-month clinical outcomes.

In most reported cases, the accessory fragment is located near the posterior talar process and may involve the subtalar joint, resulting in ankle pain, swelling, and limited range of motion^[^4,5^]^. Diagnosing a bipartite talus can be challenging because it may resemble other conditions, such as a nonunion fracture, an accessory ossicle, post-traumatic pseudarthrosis, or an atypical os trigonum. Therefore, advanced imaging modalities, including CT and MRI, are often required to better characterize the fragment, assess joint involvement, and evaluate associated degenerative changes^[^3,6^]^.

Lateral talar involvement in bipartition is extremely uncommon. A lateral fragment is clinically important because of its proximity to the lateral ankle ligament complex, particularly the anterior talofibular ligament (ATFL). Given the limited knowledge on this subject, we report a rare case of a bipartite talus located at the lateral ankle that was successfully treated with arthroscopic resection and ATFL reattachment. To our knowledge, no previous cases of bipartite talus involving the lateral ankle have been reported from Indonesia. This study has been reported in accordance with the SCARE 2025 standards[7].

## Case presentation

A 42-year-old man presented with chronic pain (VAS 7) over the lateral aspect of the left ankle, associated with a palpable, hard mass that had been present for many years. The symptoms had progressively worsened over the preceding 2 years, particularly during prolonged walking, standing, and physical activity. His occupation involved moderate daily physical activity, which aggravated the symptoms. The patient did not notice any significant increase in the size of the bony prominence over time. There was no history of acute trauma, recurrent ankle sprains, or infection. The patient had not undergone any conservative treatment, including medication or injections. The patient’s functional assessment demonstrated a FAAM ADL (Activities of Daily Living) score of 69/84 (82%) and a FAAM Sports score of 20/32 (63%).

Physical examination revealed a firm bony prominence over the lateral ankle with localized tenderness on palpation. Ankle range of motion was largely preserved, although slight pain was noted at end range of motion. Neurovascular examination was normal.

Radiographs revealed a bony prominence on the lateral aspect of the talus (Fig. 1). CT scans demonstrated a well-corticated accessory bony fragment located on the lateral aspect of the talus, separated from the main talar body by a distinct cleft (Fig. 2), consistent with a bipartite talus.

**Figure 1.**
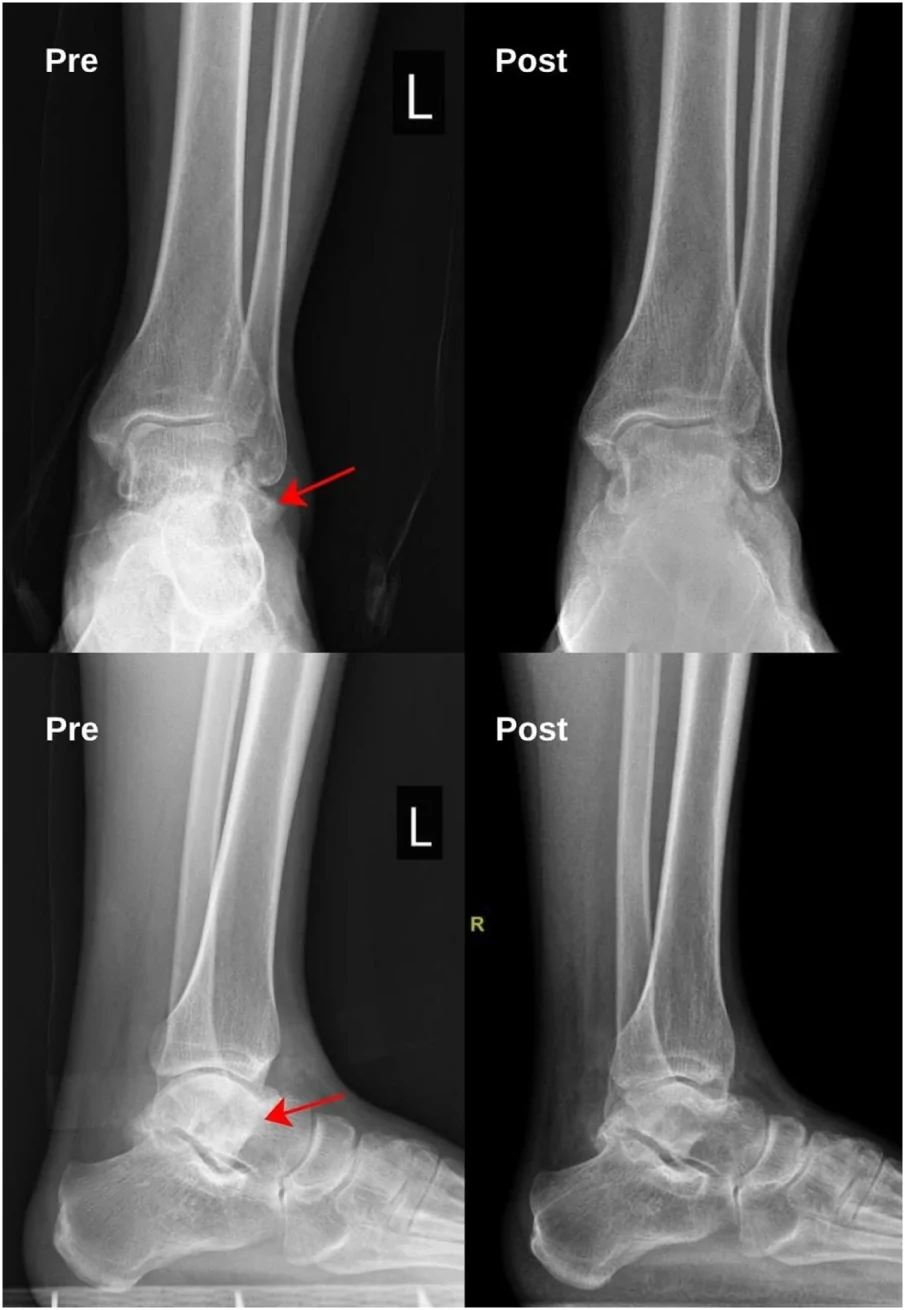
Comparative preoperative and 18-month postoperative radiographs of the left ankle, including anteroposterior (AP) and lateral views, that demonstrate a well-corticated accessory osseous fragment at the lateral aspect of the talus preoperatively (red arrow) and complete resection of the bipartite talar fragment postoperatively, without a residual osseous fragment.

**Figure 2.**
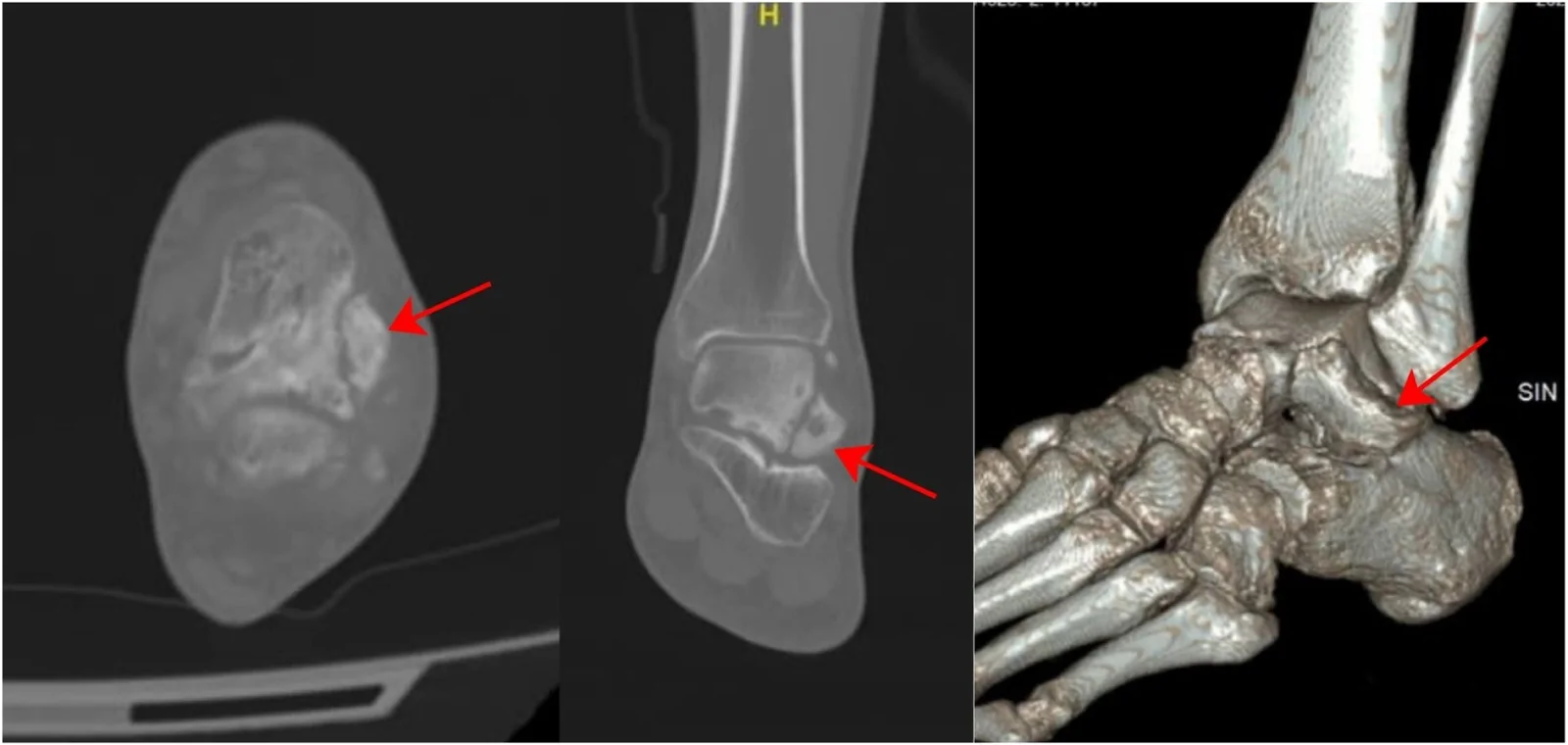
Preoperative CT of the left ankle demonstrates a distinct cleft between the talus and the accessory fragment (red arrows).

Surgical intervention was performed because of persistent pain, mechanical irritation, and functional limitation affecting daily activities. The patient underwent anterior ankle arthroscopy for resection of the accessory talar fragment (Fig. 3). The patient was placed in the supine position with a tourniquet applied to the thigh. Standard anteromedial (AM) and anterolateral (AL) portals were established. A joint evaluation was performed, and a large bipartite talar fragment was identified on the lateral side (Fig. 3A). The fragment was then divided into smaller pieces with a small osteotome and extracted through the AL portal (Fig. 3B).

**Figure 3.**
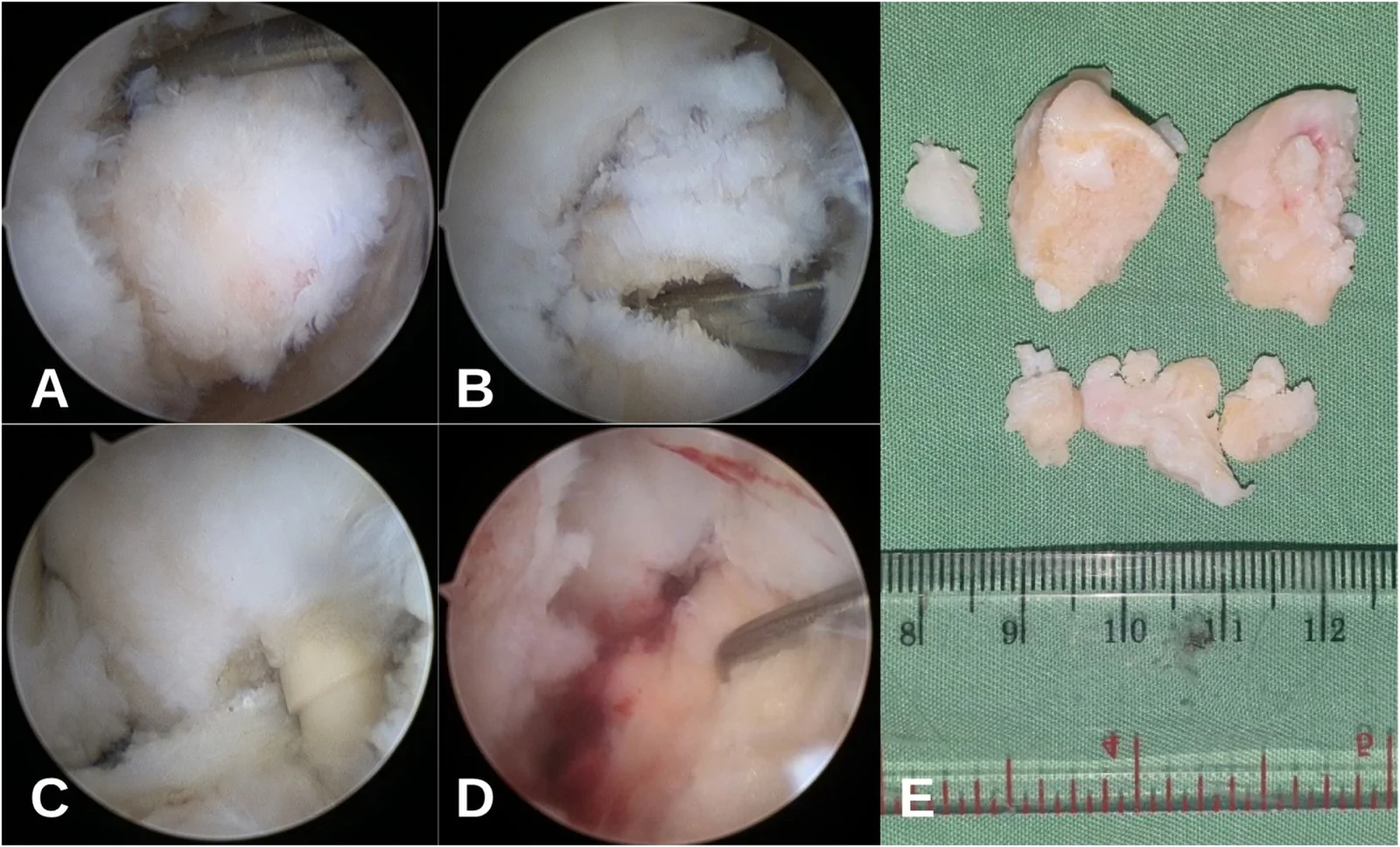
Arthroscopic and intraoperative findings. (A) Arthroscopic view of the bipartite talar fragment located at the lateral aspect of the talus. (B) The fragment was divided into smaller pieces prior to extraction using a small osteotome. (C) Arthroscopic Broström repair demonstrating suture anchor placement for anterior talofibular ligament (ATFL) reconstruction. (D) Completion of arthroscopic ATFL capsuloligamentous repair with suture fixation restores the lateral ankle ligament complex. (E) Gross appearance of the resected bipartite talar fragments following arthroscopic excision.

An accessory anterolateral (AAL) portal was created to allow a favorable working angle for anchor insertion and suture management. A single 2.9-mm all-suture anchor (Doratek, Turkey) was placed in the anterolateral gutter at the fibular footprint of the ATFL origin (Fig. 3C). Using a suture-passing device introduced through the AAL portal, the residual ATFL–capsular tissue was captured together with the inferior extensor retinaculum in a horizontal mattress suture configuration. The sutures were tensioned and secured with the ankle held in a neutral position and slight eversion to advance and imbricate the ATFL–capsular complex over the fibular footprint, thereby shortening the redundant ligament-capsule tissue and restoring native tension. Following repair, ankle stability was confirmed by the absence of excessive anterior translation or inversion of the talus. Adequate tension was achieved (Fig. 3D), and stability was confirmed again at the end of the procedure. The surgery was completed without complications.

Postoperatively, the ankle was immobilized in a neutral position using a short leg splint or removable walking boot for 2 weeks, during which non–weight-bearing ambulation with crutches was maintained. After suture removal, the patient transitioned to an ankle brace, and active range-of-motion exercises and weight-bearing as tolerated were initiated for the following 2–4 weeks. Between 6 and 8 weeks postoperatively, the ankle brace was used intermittently, primarily during exercise. From 8 to 12 weeks, the patient wore regular shoes and progressed with muscle strengthening and balance training. Sport-specific rehabilitation was initiated after 12 weeks. During follow-up, the patient reported gradual symptomatic improvement, including reduced pain and improved ankle range of motion, without any postoperative complications. Furthermore, no clinical evidence of recurrent ankle instability was observed.

At the 18-month follow-up, the patient had resumed normal daily activities with mild residual discomfort and marked symptomatic improvement. Physical examination demonstrated no tenderness over the lateral ankle, with a stable ankle, as evidenced by negative anterior drawer and talar tilt tests. Ankle range of motion was full without pain at the end of motion. The VAS pain score improved from 7/10 preoperatively to 2/10. Follow-up radiographs of the left ankle demonstrated complete removal of the fragment (Fig. 1). Functional outcomes also improved, with an FAAM ADL score of 76/84 (90%) and an FAAM Sports score of 29/32 (91%). From the patient’s perspective, he was satisfied with the surgical outcome, reporting substantial pain relief and a successful return to normal daily activities without significant functional limitations.

## Discussion

Bipartite talus is a rare congenital anomaly characterized by two non-fused fragments of the talus. Its true prevalence remains unknown due to the limited number of reported cases in the literature^[^8,9^]^. In a radiological study of 6,145 feet, tarsal bipartitism was identified in 15 patients (0.32%), with only one case of talus bipartitism (0.02%)[4]. Most reported cases of talus bipartitus involve the posterior aspect of the talus, particularly the posterolateral or posteromedial tubercles, often extending into the subtalar joint[2].

Unlike previous reports involving the posterior talus, the present case demonstrated an unusual lateral talar location associated with a palpable bony prominence over the lateral ankle. This atypical presentation contributed to chronic lateral ankle pain, mechanical irritation, and functional limitation during daily activities. Another distinctive feature of this case was the attachment of the ATFL to the bipartite talar fragment. Consequently, simple fragment excision alone would have disrupted the integrity of the lateral ligament complex, resulting in postoperative ankle instability.

Diagnosing a bipartite talus can be challenging because it may mimic several conditions, including chronic fracture nonunion, talar-sided ATFL avulsion injury, accessory ossicles, or fractures of the lateral talar process[2]. In the present case, the lateral location of the fragment and its attachment to the ATFL particularly raised the differential diagnosis of a chronic avulsion nonunion. However, several clinical and radiological findings favored a congenital bipartite variant rather than a post-traumatic lesion. The patient had no history of acute trauma or recurrent ankle sprain, and the bony prominence had been present for many years without interval enlargement. CT imaging demonstrated a well-corticated fragment with smooth sclerotic margins, a distinct cleft separating the fragment from the talar body, and no evidence of reactive bone changes such as callus formation or irregular fracture surfaces. These findings supported a chronic developmental anomaly rather than fracture nonunion[10]. Although an accessory ossicle was also considered, the fragment’s unusually large size and lateral talar origin were atypical for commonly recognized lateral ankle ossicles, such as os peroneum or os calcaneus secundarius, which are generally smaller and more rounded in morphology^[^11,12^]^.

Surgical intervention remains the definitive treatment in symptomatic cases[13]. In the present case, surgery was selected because the patient had chronic activity-related lateral ankle pain with a palpable bony prominence, and imaging demonstrated a large, well-corticated fragment that was likely contributing to mechanical irritation. Arthroscopic excision was chosen because it allowed direct visualization of the intra-articular and periarticular pathology, precise fragment removal, and minimal soft-tissue disruption compared with an open approach[6]. Intraoperatively, the ATFL was found to be attached to the bipartite talar fragment. Therefore, fragment excision alone could have compromised lateral ankle stability. For this reason, arthroscopic Broström-type capsuloligamentous repair using a suture anchor was performed to restore the ATFL–capsular restraint and to augment stability through incorporation of the joint capsule and inferior extensor retinaculum. This combined arthroscopic approach allowed both the removal of the symptomatic fragment and the restoration of lateral ankle stability, resulting in satisfactory clinical improvement[14].

This study has several limitations. First, this report describes a single case, which limits generalizability and prevents broader conclusions regarding the optimal management of bipartite talus. In addition, the diagnosis was based on the clinical history and characteristic radiographic and CT findings because MRI evaluation and histopathological examination of the excised fragment were not performed. Consequently, the diagnosis cannot be considered histologically or MRI-confirmed, which may reduce diagnostic certainty. Furthermore, the follow-up period was limited to 18 months, and longer-term outcomes remain unknown.

## Conclusion

This case highlights a rare presentation of a symptomatic lateral bipartite talus with attachment of the ATFL to the accessory fragment. CT imaging was useful in defining the chronic, well-corticated fragment and differentiating it from an acute fracture. Arthroscopic excision combined with an ATFL capsuloligamentous repair was feasible and resulted in favorable clinical and radiographic outcomes at 18 months. Surgeons should recognize that lateral talar fragments may involve the lateral ligament complex, and ligament repair may be necessary after fragment excision.

## Data Availability

Not applicable. No datasets were generated or analyzed during this study.

## References

[1] Bastawi R. Bipartite navicular bone: spectrum of imaging findings of uncommon anatomic variant frequently inducing mid-tarsal osteoarthrosis. Egypt J Radiol Nucl Med 2025;56:25.

[2] Zwiers R de Leeuw PAJ Kerkhoffs GMMJ. Talus bipartitus: a systematic review and report of two cases with arthroscopic treatment. Knee Surg Sports Traumatol Arthrosc 2018;26:2131–41. 10.1007/s00167-017-4613-8 PMC6061450 28660439

[3] Xiong X Chen M Lin G.. Arthroscopic surgery for the treatment of a geriatric bipartite talus: a case report. Medicine (Madr) 2026;105:e47195. 10.1097/MD.0000000000047195 PMC12794998 41517783

[4] Koroglu M Karakaplan M Ozdes HU. Bipartitism in tarsal bones: a retrospective analysis of clinical and radiological features. Ann Med Res 2024;31:747–52.

[5] Torres A Paulus M. Tarsal tunnel syndrome associated with a bipartite talus. JAAOS Global Res Rev 2024;8:e2300147. 10.5435/JAAOSGlobal-D-23-00147 PMC10849304 38323932

[6] de Oliveira Junior AS Moreira Mendes AA Saito GH. Bipartite talus: arthroscopic resection – case report. Tech Foot Ankle Surg 2018. doi:10.1097/BTF.0000000000000178.

[7] Kerwan A Al-jabir A Mathew G. Revised surgical CAse REport (SCARE) guideline: an update for the Age of Artificial Intelligence. PJS 2025. doi:10.70389/PJS.100079

[8] Tsuruta T Shiokawa Y Kato A. Radiological study of the accessory skeletal elements in the foot and ankle (author’s transl). Nihon Seikeigeka Gakkai Zasshi 1981;55:357–70. 7276670

[9] Schreiber A Differding P Zollinger H. Talus partitus. A case report. J Bone Joint Surg Br Vol 1985;67:430–31. 10.1302/0301-620X.67B3.3997955 3997955

[10] Agharkar Y Saravanan S. Non-union of Talus Fracture in a 34-Year-Old Male Managed with Open Reduction and Internal Fixation Using Cancellous Cannulated Screws: a Case Report. J Orthop Case Rep 2025;15:200–03. 10.13107/jocr.2025.v15.i10.6208 PMC12514930 41078696

[11] Kalbouneh H Alajoulin O Shawaqfeh J.. Accessory ossicles in the region of the foot and ankle: an epidemiologic survey in a jordanian population. Medicina (B Aires) 2021;57:1178. 10.3390/medicina57111178 PMC8618233 34833396

[12] Moon J Graham R Kushner D. Symptomatic accessory ossicles of the foot and ankle. Curr Probl Diagn Radiol 2023;52:300–11. 10.1067/j.cpradiol.2023.03.009 37085336

[13] Mann HA Myerson MS. Treatment of posterior ankle pain by excision of a bipartite talar fragment. J Bone Joint Surg Br Vol 2010. doi:10.1302/0301-620x.92b7.23465 20595113

[14] Guyonnet C Vieira TD Wackenheim FL. Arthroscopic modified broström repair with suture-tape augmentation of the calcaneofibular ligament for lateral ankle instability. Arthrosc Tech 2024;13:102887. 10.1016/j.eats.2023.11.015 PMC10995735 38584641

